# Levodopa Plus Occlusion Therapy versus Occlusion Therapy Alone for Children with Anisometropic Amblyopia

**DOI:** 10.18502/jovr.v14i4.5451

**Published:** 2019-10-24

**Authors:** Majid Farvardin, Mohammad Reza Khalili, Mehdi Behnia

**Affiliations:** ^1^Poostchi Ophthalmology Research Center, Shiraz University of Medical Sciences, Shiraz, Iran; ^2^Noor Ophthalmology Research Center, Noor Eye Hospital, Tehran, Iran

**Keywords:** Amblyopia, Anisometropic, Levodopa, Occlusion Therapy

## Abstract

**Purpose:**

This study aimed to compare the effects of short-term administration of levodopa plus occlusion therapy versus occlusion therapy alone in preschool children with hyperopic anisometropic amblyopia.

**Methods:**

This comparative interventional study included 40 eligible preschool children aged 6 to 7 years with hyperopic anisometropic amblyopia. The primary outcome measure was the logarithm of the minimum angle of resolution (logMAR) best-corrected visual acuity recorded at baseline, 3 weeks after the treatment initiation and 12 weeks after the treatment termination. The results were compared between the two groups.

**Results:**

No statistically significant intergroup difference was observed in baseline logMAR visual acuities (*P* = 0.92). The mean logMAR visual acuities of the amblyopic eyes were significantly better in both groups three weeks after the treatment initiation than the baseline (*P* < 0.01 in both groups). At 12 weeks after treatment termination, the logMAR visual acuities of the amblyopic eyes were significantly better than the baseline values (*P* < 0.001 in the placebo group and *P* = 0.09 in the levodopa group). Intergroup comparisons revealed no statistically significant difference in visual acuities 3 weeks after the treatment initiation (*P* = 0.11) and 12 weeks after the treatment termination (*P*=0.10). Twelve weeks after the treatment termination, visual acuities regressed 0.037 logMAR in the placebo group and 0.042 logMAR in the levodopa group. These regression rates were not significantly different (*P* = 0.89).

**Conclusion:**

The results of this study provide evidence that adding short-term administration of levodopa to occlusion therapy in hyperopic anisometropic amblyopia offers no additional benefit in visual outcomes and provides no advantage in terms of the regression rate.

##  INTRODUCTION

For more than 200 years, the standard treatment for amblyopia has been occlusion of the dominant eye.^[1]^ However, noncompliance is common because of cosmetic dissatisfaction and interference of occlusion with daily activities.^[2,3]^ Therefore, developing a treatment modality for amblyopia with improved efficacy, shorter duration, and better compliance has been a research goal. In the past years, several studies have evaluated the effects of levodopa on visual function in children with amblyopia.^[4,5,6,7,8,9,10,11,12]^ Levodopa is a precursor of dopamine, a neurotransmitter in the brain and retina. The effects of dopamine in the management of amblyopia are explained by its neuromodulator and neurotransmitter roles in the retina and central visual pathway. Studies have suggested that increased dopamine levels lead to a reduction in the size of the receptive field in the retina, thereby improving visual function.^[8,9,13]^ In addition, studies have hypothesized that increased dopamine levels reduce the size of the suppression scotoma. Measurement via functional magnetic resonance imaging has shown that dopamine changes the volume of cortical activation even after a single-dose administration.^[14,15]^

Peripheral decarboxylase inhibitors, such as carbidopa or benserazide, prevent the breakdown of levodopa at peripheral sites and allow more levodopa to cross the blood–brain barrier, where it can have central effects.^[16,17]^ Several studies on the influence of levodopa on visual function in amblyopia have used levodopa without occlusion of the dominant eye,^[4,7,8]^ whereas some studies have combined levodopa with occlusion of the dominant eye.^[5,6,10,11,12]^ The results of studies comparing the effects of levodopa plus occlusion therapy and occlusion therapy alone in amblyopia are controversial, and a debate persists whether augmenting occlusion therapy with a pharmacologic agent such as levodopa could affect the management of patients with amblyopia. In a single-dose study, and in another three-week longitudinal study, Leguire et al demonstrated that levodopa combined with occlusion improved the visual acuity in children with amblyopia to a greater extent than did placebo combined with occlusion.^[5,6]^ However, in children with anisometropic and strabismic amblyopia, Bhartiya et al showed that levodopa supplementation has no additional benefit over occlusion therapy alone.^[12]^

Therefore, the present study aimed to assess the effectiveness of short-term administration of levodopa combined with standard occlusion treatment on the visual acuities of preschool children with hyperopic anisometropic amblyopia, and to compare it to that of occlusion therapy alone.

##  METHODS

###  Study Design and Participants

**Figure 1:**
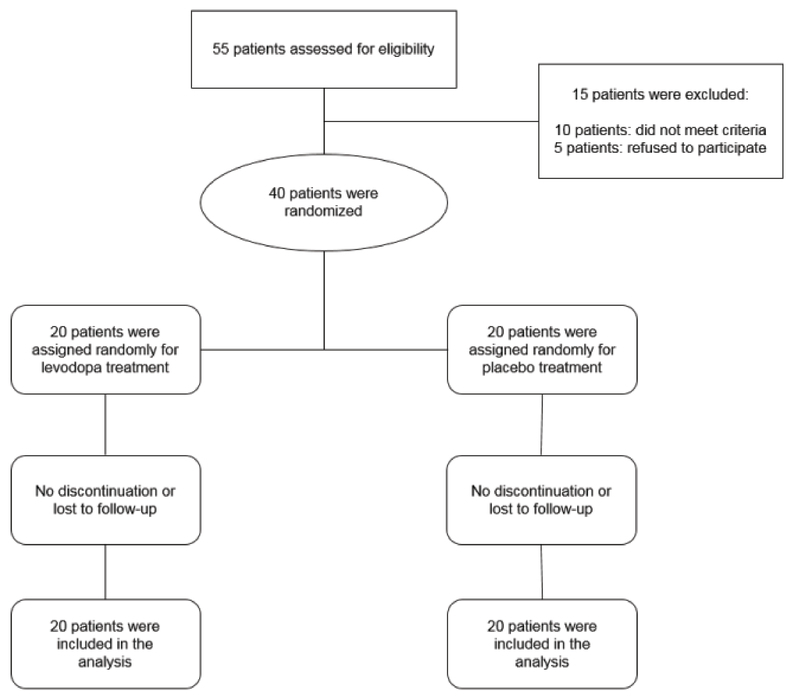
Flow diagram outlining the enrollment of the amblyopic children into the study.

In this comparative interventional study, we included 40 eligible patients with hyperopic anisometropic amblyopia. The patients were preschool children aged 6 to 7 years with amblyopia caused by hyperopic anisometropia [Table 1 and Figure 1]. All patients were selected among the preschool children referred from the national amblyopia screening program to our strabismus and pediatric ophthalmology clinic affiliated to the Shiraz University of Medical Sciences because of decreased visual acuity. The inclusion criteria were as follows: patients with hyperopic anisometropia (with more than 1.5 diopters of difference between the two eyes) and the logarithm of the minimum angle of resolution (logMAR) best-corrected visual acuity (BCVA) worse than 0.2 log-unit in the amblyopic eye or at least a 2-line difference in BCVA between the two eyes. The eye with worse BCVA was included in the study. At an alpha level of 0.05, 18 patients were required in each group to find a difference of 0.1 logMAR visual acuity between the treatment and placebo groups. Considering a 10% possibility of non-adherence, 40 patients were included.

**Table 1:** Absolute refractive errors of both groups and differences in refractive errors (mean and standard deviation of the spherical equivalent of anisometropia in both groups)


	**Placebo**	**Levodopa**	**** ***P*** **-value**
Normal eye	1.99 ± 0.69	1.69 ± 0.77	0.28
Amblyopic eye	4.49 ± 0.59	4.29 ± 0.58	0.94
Difference	2.49 ± 0.82	2.61 ± 0.96	0.49
	
	

Patients with strabismic amblyopia, other types of anisometropic amblyopia and deprivation amblyopia, and patients with a history of previous amblyopia therapy were excluded from the study. We also excluded patients with neurologic and cognitive disorders, including cerebral palsy, autism spectrum disorders, and attention deficit hyperactive disorder, as well as those with any systemic disorders such as diabetes, thyroid disease, and rheumatologic disease. Eyes with a history or objective sign of trauma, previous intraocular surgery, uveitis, corneal opacity, or any ocular disorder other than amblyopia and hyperopic anisometropia were also excluded.

All eligible patients underwent a complete ocular examination, including visual acuity measurement, slit-lamp examination, dilated fundus examination, ocular motility test, and appropriate strabismus tests (including prism-cover test and modified Krimsky test). We used the Snellen E-chart, and Snellen visual acuity was measured under standardized lighting conditions in the same room with the same projector unit and at a viewing distance of 6 m. Refraction was measured under cycloplegia with 1% cyclopentolate eye drops (cycloplegic refraction). We assessed the best subjective refraction, measured visual acuity by using optical correction, and registered the BCVA.

All interventions were conducted according to the tenets of the Declaration of Helsinki, and the study was approved by the Ethics Committee of our institution. Signed informed consent was obtained from at least one parent, and assent was obtained from each child at the beginning of the study.

Eligible preschool children were divided into two groups of 20 patients each. In the first group, part-time occlusion therapy was administered 3 h/day combined with an oral placebo, and in the second group, part-time occlusion therapy was administered 3 h/day with levodopa. The dosage of levodopa was based on each patient's body weight; each patient received 6 mg/kg as a loading dose. Thereafter, each patient received 2 mg/kg/day levodopa in three divided doses administered for three weeks. The Pharmacy Department of the Shiraz University of Medical Sciences prepared the levodopa-benserazide and placebo capsules. The placebo was made using the same capsules used for levodopa, but they were filled with lactose instead of the active drug. Patients were instructed to take one capsule three times/day at approximately 8-h intervals after a meal. Visual acuities were measured and recorded by an expert optometrist. Neither patients nor examiners were aware of the difference.

The patients were followed up and examined at weeks 1 and 3 during the treatment regimen and 12 weeks after the termination of all treatments. All patients were informed about the potential side effects of the drug such as nausea, vomiting, headache, dizziness, dry mouth, fatigue, nightmares, and mood changes. Parents were instructed to record the date(s) of any side effects. At each follow-up, the patients were asked whether they experienced any subjective changes or side effects. Systolic and diastolic blood pressure and pulse rate were also measured at each examination session. Laboratory tests, including complete blood count and differential, liver function tests, blood urea nitrogen (BUN), and plasma creatinine level measurements were performed at baseline, one week and three weeks after the initiation of treatment, and at three months (12 weeks) after the termination of all treatments. If a patient's laboratory test results were not within the normal limits, the patient was excluded from the study.

###  Study Outcome Measures

A change in Snellen BCVA was regarded as the primary outcome measure. The amblyopic eye was always tested first, and one varying line was presented each time to prevent memorization within a test session. The line in which the patient could read more than half of the letters was considered as the line of visual acuity. If the new glasses resulted in better BCVA, the glasses were changed, and the patient was given time to adapt to the new prescription before starting the assigned treatment. The BCVA values were converted to logMAR visual acuities for statistical purposes. The regression rate and side effects were secondary outcome measures.

###  Statistical Analysis

All statistical analyses were performed using SPSS Statistics for Windows/Macintosh, Version 17.0 (SPSS Inc., Chicago, IL). A paired two-tailed *t*-test was used to compare the logMAR visual acuities in consecutive test sessions in each group. The independent samples *t*-test was used to compare the logMAR visual acuities between the two groups. A *P* < 0.05 was considered statistically significant.

##  RESULTS

The mean (±standard deviation) age of the patients was 6.4 years (±0.416) in the levodopa group and 6.4 years (±0.447) in the placebo group. Both groups included 10 male and 10 female patients. The absolute refractive errors of both groups and the difference in refractive errors (mean and standard deviation of the spherical equivalent of anisometropia in both groups) are shown in Table 1. No statistically significant difference was observed between the groups in the spherical equivalent of anisometropia (*P* = 0.49).

The mean and standard deviation of logMAR visual acuities of the patients in both groups at each examination are represented in Table 2. No statistically significant difference was observed between the groups in the baseline visual acuities of the amblyopic eyes (*P* = 0.92). Visual acuities were significantly better at three weeks after the initiation of treatment than at the baseline in both groups (*P* = 0.002 in the placebo group and *P* = 0.007 in the levodopa group). The improvement in visual acuities in the amblyopic eyes was not significantly different between the levodopa and placebo treatment groups (*P* = 0.11). To assess the stability of the treatment effects, we repeated the examinations 12 weeks after the termination of all treatments. Visual acuities at this examination were still significantly better than the baseline visual acuities (*P* < 0.001 in the placebo group and *P* = 0.09 in the levodopa group). No statistically significant difference was observed between the visual acuities of the levodopa and placebo treatment groups (*P* = 0.10). Visual acuities regressed 0.037 logMAR in the placebo group and 0.042 logMAR in the levodopa group 12 weeks after the termination of all treatments. A comparison of the regression rates between the groups showed no statistically significant difference (*P* = 0.89).

**Table 2:** Visual acuities of patients in the placebo and levodopa groups at each examination


**Group**	**Baseline mean ± SD logMAR VA**	**Three-week mean ± SD logMAR VA**	**Fifteen-week mean ± SD logMAR VA**
Placebo group	0.425 ± 0.231	0.234 ± 0.096	0.271 ± 0.087
Levodopa group	0.432 ± 0.242	0.187 ± 0.085	0.229 ± 0.071
*P*-value	0.92	0.11	0.10
	
	
logMAR, logarithm of the minimum angle of resolution; SD, standard deviation; VA, visual acuity			

###  Compliance

All patients completed all test sessions. According to previous reports, capsule consumption compliance was determined using the number of remaining capsules at the end of the treatment period.^[10]^ Regimen capsule compliance in both groups was good, and no capsule remained at the end of the treatment.

###  Tolerance

Patients were asked, during each follow-up, whether they experienced any side effects. In the first week, four patients in the placebo group (20%) had an episode of mild nausea lasting for few hours, but it did not continue during the course of the study and did not necessitate discontinuation of the treatment. No patient reported headache, dizziness, fatigue, nightmares, and dry mouth. No patients reported side effects in the levodopa group. No significant changes were observed in systolic or diastolic blood pressures or pulse rate over the 15-week study period. Moreover, no occlusion amblyopia occurred in the fellow eyes in the control and treatment groups.

###  Laboratory Tests

A comparison of the baseline and 15-week study results revealed no changes in various laboratory tests. The complete blood count and differential results remained unchanged. No specific changes were observed in the serum BUN and creatinine levels. Moreover, no change was noted in the liver function test results.

##  DISCUSSION

The results of this placebo-controlled interventional study suggest that adding short-term administration of levodopa to the standard part-time occlusion therapy in preschool children with hyperopic anisometropic amblyopia does not yield better visual outcomes.

For this study, we selected patients with hyperopic anisometropic amblyopia, which is the most common type of anisometropic amblyopia. Other types of amblyopia, such as strabismic amblyopia and stimulus deprivation amblyopia are usually diagnosed by parents at an earlier age and, hence, treatment begins earlier. However, patients with anisometropia have normal general appearance and usually present at a later age; they are frequently diagnosed during preschool amblyopia screening programs. Thus, the problem of neglected amblyopia is of major importance in anisometropic amblyopia, and anisometropia is the most common cause of amblyopia in adults.^[3]^

Several studies on the treatment of amblyopia have shown the beneficial effects of levodopa.^[4][5,6][7][8][9,10][11]^ The beneficial effects of levodopa and benserazide in decreasing the fixation scotoma size and improving contrast sensitivity in amblyopia have already been demonstrated.^[7]^ Like carbidopa, benserazide is a peripheral decarboxylase inhibitor. Levodopa is a precursor to the neurotransmitter dopamine and is administered to increase the dopamine level in the central nervous system. However, most levodopa is decarboxylated to dopamine before it reaches the brain, and since dopamine is unable to cross the blood–brain barrier, this leads to little therapeutic effect in the brain with strong peripheral side effects. By inhibiting the aforementioned decarboxylation, benserazide allows dopamine access to the brain. In addition, the adverse effects caused by peripheral dopamine, such as vasoconstriction, nausea, and arrhythmia are minimized.

In a study on adults with amblyopia, Gottlob et al showed an improvement in visual acuity after one-week levodopa therapy.^[8]^ In a single pilot study, Leguire et al demonstrated the effects of levodopa/carbidopa on visual function.^[4]^ Based on these initial findings, other studies were designed to evaluate the effects of levodopa in human amblyopia.^[5,6][9,10][11][12]^ Some studies compared the effects of levodopa therapy alone and levodopa plus occlusion therapy on the visual function of amblyopic eyes.^[9,10]^ Other studies compared the effects of levodopa plus occlusion therapy and occlusion therapy alone in amblyopia. The results of these studies appeared to be contradictory; some studies suggested beneficial effects,^[5,6][18]^ whereas others demonstrated that this treatment was ineffective.^[12,19]^ Leguire et al, in a placebo-controlled, single-dose study and in another three-week longitudinal study on children with amblyopia, showed that levodopa combined with occlusion resulted in more improved visual acuity than did placebo combined with occlusion.^[5,6]^ Dadeya et al also compared the effects of combined levodopa and occlusion therapy to those of occlusion therapy alone in the management of patients with strabismic amblyopia; more patients in the levodopa group gained more than two lines of visual acuity.^[18]^ Contrary to these studies, in a randomized, placebo-controlled study on children with amblyopia, Bhartiya et al reported that levodopa supplementation did not have any advantage over occlusion therapy alone.^[12]^ Rashad et al also observed that in children and adults with different types of amblyopia, the mean logMAR was similar in the occlusion and levodopa enhancement groups.^[19]^ In their study, a higher percentage of strabismic amblyopia was observed in the levodopa group and a higher percentage of mixed-etiology amblyopia was noticed in the occlusion group.

Unlike the abovementioned studies, we exclusively included patients with hyperopic anisometropic amblyopia in both groups and, therefore, compared similar groups. The similar age (preschool children) in both groups in the present study also allowed for a more reliable comparison. Despite several differences among the studies, the present study findings are in agreement with the studies of Bhartiya et al and Rashad et al who showed that supplementation of occlusion therapy with levodopa offers no additional benefits in patients with amblyopia compared with occlusion therapy alone. Moreover, a randomized trial of levodopa as a treatment for residual amblyopia in older children demonstrated that oral levodopa administration while continuing patch therapy 2 h/day does not produce a clinically or statistically significant improvement in comparison with placebo administration or patching alone.^[20]^

The aim of amblyopia therapy is to improve the visual acuity of the amblyopic eye and to prevent the regression of visual acuity. Many patients with successfully treated amblyopia are known to exhibit a regression of visual acuity once standard occlusion therapy is terminated.^[2]^ Long-term follow-up of levodopa treatment in children with amblyopia has shown that the regression of visual acuity in patients receiving levodopa therapy alone was more (2.1 lines) than that in those receiving levodopa plus occlusion (1.4 lines).^[11]^ Pandey et al also showed that visual acuity regressed significantly when levodopa was not combined with occlusion therapy.^[21]^ Mohan et al demonstrated that the addition of full-time occlusion to levodopa helped maintain improved visual acuity for a longer duration than did levodopa alone.^[22]^ According to our results, visual acuities regressed 0.037 logMAR in the placebo plus occlusion treatment group and 0.042 logMAR in the levodopa plus occlusion treatment group 12 weeks after the termination of all treatments. Although the mean value of regression was less in the placebo group, a comparison of the regression rates between the groups showed no statistically significant difference. Following the termination of treatments in both groups, and when the regression of visual acuity occurred, the patients still had better visual acuities than their baseline visual acuities.

Both groups completed all test sessions and tolerated treatment well without significant side effects. Before enrollment, the parents or other caregivers were informed about the possible adverse effects of medications, such as hypotension, arrhythmias, disorientation, confusion, and extreme emotional states, particularly anxiety, insomnia, visual hallucinations, somnolence, narcolepsy, depression, and psychosis. However, these side effects are very uncommon given the short duration of study. Moreover, no significant side effect was reported by the patients in the present study. Abnormalities in laboratory tests may occur in patients receiving levodopa.^[23,24,25]^ These include elevations in liver and kidney function test parameters, such as alkaline phosphatase, serum glutamic-oxaloacetic transaminase/aspartate aminotransferase, serum glutamic-pyruvic transaminase/alanine aminotransferase, and bilirubin levels, and abnormalities in BUN and creatinine levels.^[23]^ Thrombocytopenia has also been reported. Nevertheless, in our study, no abnormality in laboratory data was detected.^[24,25]^

One limitation of our study was the small sample size. However, according to calculations, 18 patients were required in each group to find a difference of 0.1 logMAR visual acuity between the treatment and placebo groups, and considering a 10% possibility of non-adherence, we included 40 patients. In addition, the present study was performed on children with hyperopic anisometropia aged 6 to 7 years old; hence, the results cannot be generalized to other amblyopia types and to patients in other age groups.

In conclusion, the results of the present study indicated that in preschool children with hyperopic anisometropic amblyopia, short-term administration of levodopa combined with conventional occlusion therapy offered no additional benefits in visual outcomes than did occlusion therapy alone. Moreover, the combined treatment offered no advantage over the placebo treatment in terms of the regression rate. This clearly indicates the need for more studies to clarify the role of levodopa in the management of amblyopia.

##  Financial Support and Sponsorship

Nil.

##  Conflicts of Interest

There are no conflicts of interest.
